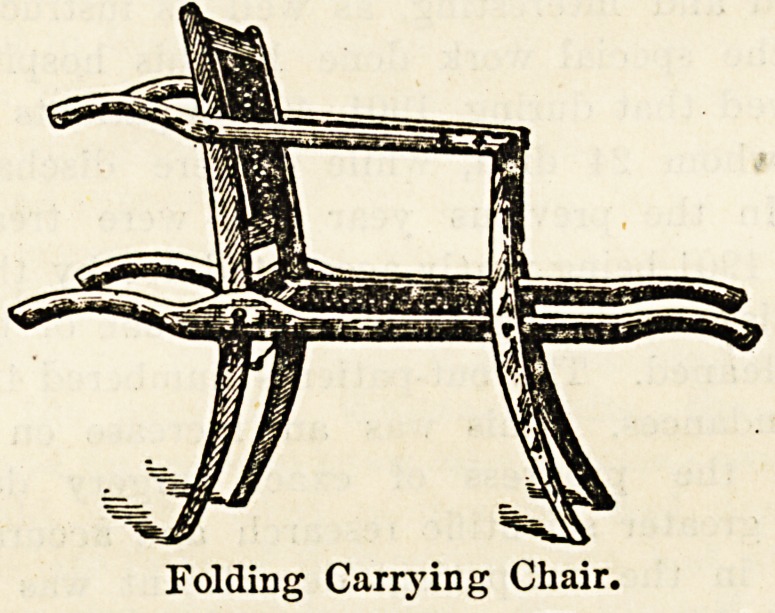# Practical Departments

**Published:** 1902-04-05

**Authors:** 


### PRACTICAL DEPARTMENTS.

### A NEW MERLIN CHAIR.

Messrs. Farmer, Lane & Co., of 77–79 New Oxford Street, London, W.C., have lately produced a new pattern of Merlin

The Merlin Chair.

chair. Hitherto, these chairs have been constructed with square backs, shaped, of course, to accommodate the human figure,

Folding Carrying Chair.

but not curved from back to front. The new chair, as may be seen by the accompanying illustration, is caned all the way round, instead of being open under the arm rests. To make the chair still more comfortable, cushions may be fitted into the back, seat, and sides. These are of hair, and make a very efficient as well as comfortable support for an invalid; or the entire chair can be upholstered if preferred. As in all these chairs, there is a sliding foot-rest in this "circular back chair," which is made in birch, walnut, mahogany, or oak. Twenty-one of these chairs have recently been supplied to a north-country hospital. The other illustration shows a caned folding carrying chair, which packs quite flat for travelling. It is light, but firm, and makes a very suitable adjunct to an invalid equipage when going abroad, etc., while for carrying a patient up and down the stairs of his own house it is equally useful. The fact that it folds up and occupies very little space enhances its value. The Bath chairs, rim-wheel chairs, walking machines, and other invalid furniture made by this firm are almost too well known to need comment; and we shall only add that the greatest care is exercised in the selection of good and well-seasoned materials, which stand the test of time. All these things are made by the firm's own workpeople, and there are no branch establishments.

## Figures and Tables

**Figure f1:**
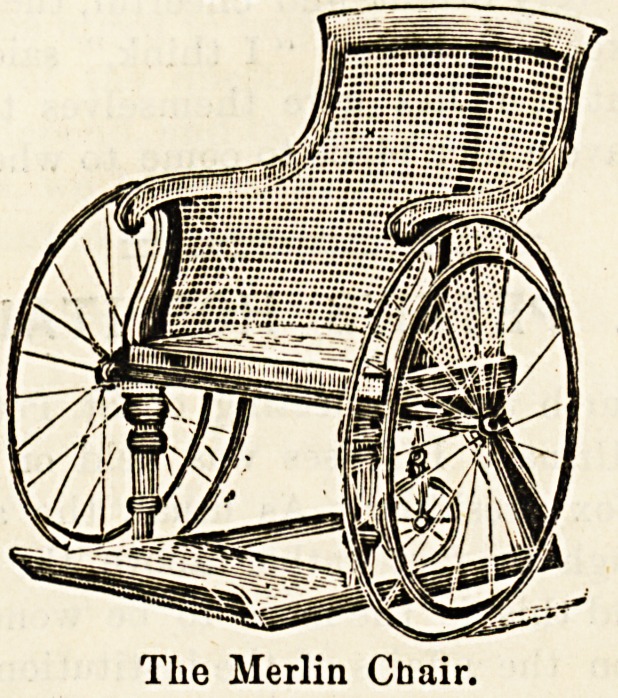


**Figure f2:**